# Approaches for Identification of HIV-1 Entry Inhibitors Targeting gp41 Pocket

**DOI:** 10.3390/v5010127

**Published:** 2013-01-11

**Authors:** Fei Yu, Lu Lu, Lanying Du, Xiaojie Zhu, Asim K. Debnath, Shibo Jiang

**Affiliations:** 1 Lindsley F. Kimball Research Institute, New York Blood Center, New York, NY 10065, USA; E-Mails: fyu2@nybloodcenter.org (F.Y.); ldu@nybloodcenter.org (L.D.); adebnath@nybloodcenter.org (A.K.D.); 2 Key Laboratory of Medical Molecular Virology of Ministries of Education and Health, Shanghai Medical College and Institute of Medical Microbiology, Fudan University, Shanghai 200032, China; E-Mails: lul@fudan.edu.cn (L.L.); 297031007@qq.com (X.Z.)

**Keywords:** HIV-1, gp41, HIV fusion/entry inhibitors, small molecule compounds, hydrophobic pocket

## Abstract

The hydrophobic pocket in the HIV-1 gp41 N-terminal heptad repeat (NHR) domain plays an important role in viral fusion and entry into the host cell, and serves as an attractive target for development of HIV-1 fusion/entry inhibitors. The peptide anti-HIV drug targeting gp41 NHR, T-20 (generic name: enfuvirtide; brand name: Fuzeon), was approved by the U.S. FDA in 2003 as the first HIV fusion/entry inhibitor for treatment of HIV/AIDS patients who fail to respond to the current antiretroviral drugs. However, because T20 lacks the pocket-binding domain (PBD), it exhibits low anti-HIV-1 activity and short half-life. Therefore, several next-generation HIV fusion inhibitory peptides with PBD have been developed. They possess longer half-life and more potent antiviral activity against a broad spectrum of HIV-1 strains, including the T-20-resistant variants. Nonetheless, the clinical application of these peptides is still limited by the lack of oral availability and the high cost of production. Thus, development of small molecule compounds targeting the gp41 pocket with oral availability has been promoted. This review describes the main approaches for identification of HIV fusion/entry inhibitors targeting the gp41 pocket and summarizes the latest progress in developing these inhibitors as a new class of anti-HIV drugs.

## 1. Introduction

The acquired immunodeficiency syndrome (AIDS) caused by human immunodeficiency virus (HIV) is still considered as one of the most life-threatening diseases. Since the beginning of the epidemic, more than 60 million people have been infected with HIV, and over 25 million have died from the disease [1]. The gradual increase in new HIV infections only exacerbates the situation. Unfortunately, no efficient vaccines against the virus are currently available. Accordingly, development of potent and affordable anti-HIV drugs is the main therapeutic thrust in treating patients with HIV infection. So far, four classes of anti-HIV drugs have been approved by U.S. Food and Drug Administration (FDA), including reverse transcriptase inhibitors (RTIs), protease inhibitors (PIs), fusion and entry inhibitors, and integrase inhibitors. In particular, HIV-1 fusion/entry inhibitors can target early steps of the HIV replication cycle, and they can be used to treat patients who fail to respond to the RTIs and PIs [2]. 

**Figure 1:**
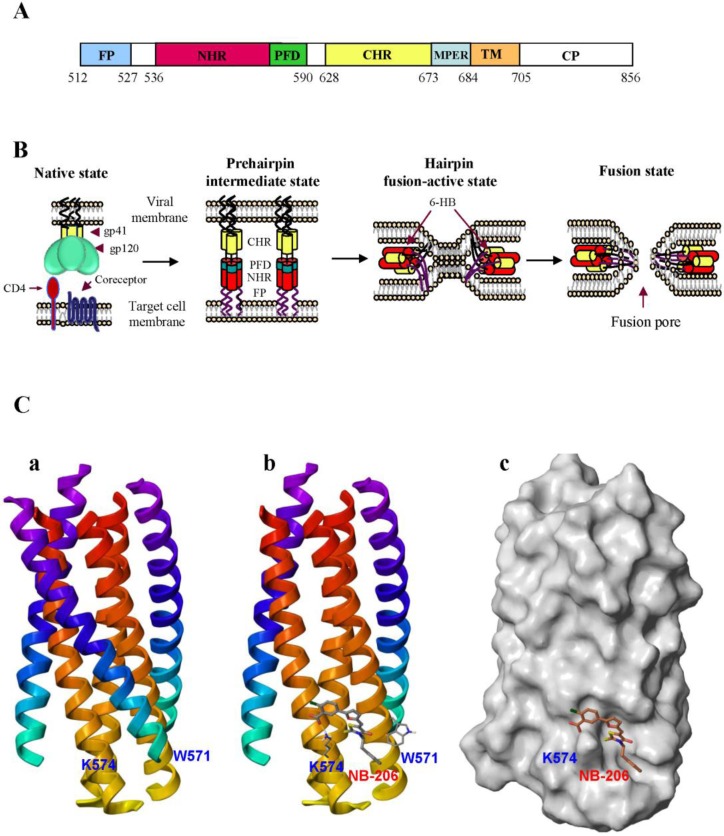
**Model of HIV-1 gp41-mediated membrane fusion and interaction of the HIV-1 entry inhibitor with the pocket in gp41. **(**A**) Schematic view of the HIV-1_HXB2 _gp41 molecule. *FP*, fusion peptide; *NHR*, N-terminal heptad repeat; *PFD*, pocket-forming domain; *CHR*, C-terminal heptad repeat; *MPER,* membrane-proximal external region; *TM*, transmembrane domain; and *CP,* cytoplasmic domain. (**B**) Model of HIV-1 gp41-mediated membrane fusion. Fusion of the HIV-1 envelope and target cell membrane is initiated by binding of the viral Env surface subunit gp120 to the cellular CD4 and then to a coreceptor (CCR5 or CXCR4) on the target cell. The Env transmembrane subunit gp41 changes conformation by inserting the FP into the target cell membrane and forming 6-HB between the viral gp41 NHR and CHR regions, bringing the viral and target cell membranes into close proximity for fusion (**C)** The crystal structure of the gp41 6-HB and docking of NB-206 in the gp41 hydrophobic pocket cavity. (**a**) Side view of the gp41 6-HB core structure formed by the N-peptide, N36, and C-peptide, C34. (**b**) Stereo view of NB-206 docked in the hydrophobic pocket showing the possible interactions with the neighboring hydrophobic and charged residue K574. (**c**) Surface representation of the gp41 core (with one C-peptide removed) with bound ligand NB-206, which docks inside the cavity with the negatively charged COOH group pointing towards the positively charged side chain of K574.

HIV type 1 (HIV-1) enters into a target cell by membrane fusion, which is mediated by the viral envelope glycoprotein (Env) transmembrane subunit gp41. HIV-1 gp41 is composed of 345 amino acid residues, corresponding to the sequence of 512–856 of the HXB2 gp160. It consists of an ectodomain (residues 512–683), a transmembrane domain (TM, residues 684–704) and a cytoplasmic domain (CP, residues 705–856). The ectodomain of HIV gp41 contains three important functional regions: the fusion peptide (FP, residues 512–527), the N-terminal heptad repeat (NHR, residues 542–592), and the C-terminal heptad repeat (CHR, residues 623–663) (Figure 1A [3]. 

Fusion of the HIV-1 envelope and target cell membranes is initiated by binding of the viral Env surface subunit gp120 to the cellular CD4, and then to a coreceptor (CCR5 or CXCR4) on the target cell. The Env transmembrane subunit gp41 changes conformation by inserting the FP into the target cell membrane. Three NHR domains form the central trimeric coiled coils that have three hydrophobic grooves, each one containing a deep hydrophobic pocket. Three CHR helices then pack into the grooves on the NHR-trimer in an antiparallel manner to form a six-helix bundle (6-HB) core, which brings the viral and target cell membranes into close proximity for fusion (Figure 1B) [4,5,6,7]. The HIV-1 gp41 hydrophobic pocket plays a critical role in stabilizing gp41 6-HB core formation and gp41-mediated membrane fusion [8,9]. Binding of a molecule to the pocket may block HIV-1 fusion with the host cell, suggesting that this pocket is an important target for development of HIV-1 entry inhibitors. Here we review the progress thus far made in developing peptide- and small molecule compound-based HIV fusion/entry inhibitors targeting the HIV-1 gp41 pocket.

## 2. Development of HIV Entry Inhibitor Peptides Targeting to gp41

The peptides derived from the gp41 NHR and CHR regions, designated N- and C-peptides, respectively, can interact with the counterpart region of the viral gp41 to form heterologous 6-HB, thus blocking viral gp41-mediated membrane fusion. To evaluate the anti-HIV-1 activity and determine the mechanisms of action of the N- and C-peptides, a series of biophysical and virological assays have been developed.

### 2.1. Development of Biophysical Methods for Identification of Inhibitors Against gp41 6-HB Formation

Sedimentation equilibrium by analytical ultracentrifugation was first utilized by Lu and colleagues for analysis of the oligomeric state of N- and C-peptides and their complexes by calculating their molecular weights, based on the slopes of the linear curves and residues, and deducing their structures [10]. They found that mixing the N-peptide N51 and C-peptide C43 resulted in the formation of a trimer of heterodimers (or 6-HB), which consists of three molecules each of the N- and C-peptides. Using similar methods, they also determined the formation of 6-HB between N36 and C34 [11]. Although this method can be used to detect the inhibitory activity of a peptide to block 6-HB formation, most biological laboratories do not have access to the very costly analytical ultracentrifuge equipment. 

Circular dichroism (CD) spectroscopy is a valuable technique for detecting conformational changes in peptides or proteins. We and others have used a CD spectrometer to monitor the conformational changes of the N- and C-peptides when they are mixed [10,12]. We have observed that the individual N36 and C34 peptides do not adapt to a stable conformation, as shown by the distinctive CD spectra of random coils, while the equimolar mixture of the two peptides does exhibit the formation of a helical complex, most likely the 6-HB, as characterized by the saddle-shaped negative peak in the far UV region of the CD spectrum and the significant increase of molar ellipticity at 222 nm [13]. In the presence of an HIV fusion inhibitor targeting gp41, such as NB-2, the α-helicity of the N36/C34 mixture was significantly decreased, and the 6-HB conformation was disrupted [14]. 

**Figure 2:**
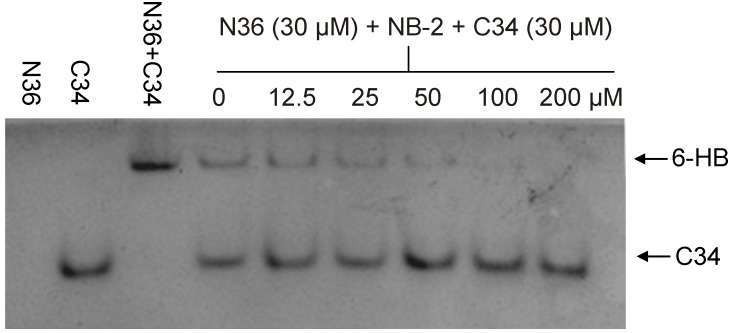
**NB-2 inhibits 6-HB formation in a dose-dependent manner, as assessed by N-PAGE. **NB-2 at different concentration was mixed with N36 at 37 °C for 30 min before addition of C34. After incubation for another 30 min, the mixture was analyzed by N-PAGE. As shown in this figure, N36 showed no band (lane 1), C34 showed a single band in the lower portion of the gel (lane 2), and the mixture of N36 and C34 showed a band in the upper portion of the gel corresponding to the band for 6-HB (lane 3). In the presence of increasing concentration of NB-2, the bands of the 6-HB formed between N36 and C34 (upper bands in lanes 4–9) become weaker and weaker than that without NB-2 (upper band in lane 3).

Because of the above problems, we have optimized a very convenient method known as native polyacrylamide gel electrophoresis (N-PAGE) to detect 6-HB formation, and this method can be performed in all biology laboratories. We have demonstrated that N-peptide N36 shows no band since N-peptides generally carry net positive charges, thus migrating up and off the gel. The C-peptide C34, however, displayed a band in the gel’s lower part, while the mixture of N36 and C34 shows a major band in the upper part of the gel, which corresponds to that of the 6-HB, and a minor band in the lower part at the same position as that of the individual C34 peptide [12]. The addition of NB-2, an HIV-1 entry inhibitor targeting gp41, to the N- and C-peptide mixture resulted in the disappearance of the upper band corresponding to that of the 6-HB (Figure 2) [14,15], suggesting that N-PAGE can be used for the identification of the inhibitors against the gp41 6-HB formation. 

Later, we further developed a fluorescence N-PAGE (FN-PAGE) using FITC-conjugated C34 to replace C34 that is used in N-PAGE. Immediately after electrophoresis, fluorescence bands in the gel could be imaged by a fluorescence imaging system using a transillumination UV light source with excitation wavelength at 302 nm and a fluorescence filter with emission wavelength at 520 nm [12]. By removing the Coomassie blue staining step, FN-PAGE is more convenient and rapid than N-PAGE, and it is more sensitive than N-PAGE for detecting HIV fusion inhibitors. 

### 2.2. Peptides Targeting gp41 NHR-Trimer and Its Pocket

In the early 1990s, we and others discovered several C-peptides with HIV-1 fusion inhibitory activity at nM levels, including SJ-2176 (residues 630–659) [16,17], DP178 (also known as T-20, residues 638–673) [18], and C34 (residues 628–661) [11]. Clinical trials indicate that T-20 can significantly decrease viral load and increase CD4 cell counts after 24 weeks of treatment [19,20]. In 2003, T-20 (generic name: enfuvirtide; brand name: Fuzeon) was approved by the U.S. FDA for use as the first HIV-1 fusion inhibitor [21,22]. However, the clinical application of T-20 is limited because it lacks the pocket-binding domain (PBD), resulting in reduced potency and a short half-life (about 3.8 h). Consequently, T-20 must be injected subcutaneously twice daily (90 mg per dose), resulting in high cost to the patients and serious local injection reactions. Furthermore, T-20 could rapidly induce HIV-1 variants with strong drug resistance in patients [23,24,25].

To overcome the above shortcomings, T-1249, a synthetic 39-residue peptide, was designed as the second-generation HIV fusion inhibitor by adding the PBD to the N-terminus of the T-20-based peptide. The *in vitro* and *in vivo* studies have demonstrated that T1249 is effective against T-20-resistant HIV-1 strains, and it exhibits a longer half-life than T-20 in non-human primates. In addition, T-1249 was well tolerated without dose-limiting toxicity in phase I/II clinical trials. Unfortunately, further development of T1249 was terminated because of insufficient improvements on the bioavailability and tolerability characteristics of T-20 [26,27]. Based on the results of T1249, researchers at Trimeris further developed a series of peptides containing PBD, such as T2635 and T1144, as the third-generation peptide fusion inhibitors, which showed longer half-life, more potent antiviral activity against a broad spectrum of HIV-1 strains, including T-20-resistant variants, and a stronger genetic barrier to drug resistance [28].

Clinical application of T-20 has shown that it can quickly induce mutations in the GIV motif (residues 547–556) in the viral gp41 NHR domain, resulting in high resistance [29]. Since T-1249 and other PBD-containing C-peptides also contain the GIV motif-binding domain, they are less susceptible to T-20-resistant HIV-1 strains [30]. To overcome this problem, we designed two peptides, CP32 and CP32M, which contain only the PBD, but no motif-binding domain. We found that these peptides are highly effective against T-20-resistant strains [31,32]. However, the viruses with mutations in the gp41 pocket region are resistant to CP32M, confirming that the C-peptides with PBD do indeed target the gp41 hydrophobic pocket [33]. 

Sifuvirtide, a C-peptide also containing PBD, was designed on the basis of the structure of C34 and the three-dimensional structure of the HIV-1 gp41 fusogenic core conformation [34,35,36]. It shows much higher potency, longer half-life and better drug resistance than T-20. The data from the phase IIb clinical trial in China indicate that Sifuvirtide could substantially improve efficacy over traditional treatment and the rate of undetectable viral loads, while the rate of CD4 cell count increments for the Sifuvirtide group was 59%, which is about 89% better than that for the control group. Furthermore, the injection site reaction is 7% for Sifuvirtide compared to 98% for T-20.

### 2.3. Rational Design of Peptides Targeting gp41 CHR-Helices

Unlike the C-peptides, most of the N-peptides, such as DP107 (also known as T21, residues 553–590) [37] and N36 (residues 546–581) [11] inhibit HIV-1 fusion by interacting with the viral gp41 CHR-helices to form heterologous 6-HB core [38]. However, their anti-HIV-1 activity is generally 100- to 1000-fold lower than the C-peptides [39], possibly because most N-peptides have a tendency to aggregate under physiological condition [10].

To solve this problem, a polypeptide, named 5-Helix, was designed as an HIV-1 fusion inhibitor targeting the gp41 CHR region [40]. Five-Helix was designed by linking three N-peptides (N40, residues 543-582) and two C-peptides (C38, residues 625-662) with a GGSGG linker, forming a single polypeptide. Unlike the 6-HB, 5-Helix contains five of six α-helical coils and exposes one of the three grooves to attract a C-helix or C-peptide to fill in the gap and prevent 6-HB core formation, thus blocking HIV-1-mediated membrane fusion. It inhibits HIV-1 fusion and replication at low nanomolar level; thus, it is much more potent than most CHR-targeting N-peptides, probably because 5-helix is well folded, soluble and extremely stable. 

Although most N-peptides inhibit HIV-1 entry by targeting the gp41 CHR domain, some mutant N-peptides, such as N36^Mut(e,g)^, inhibit viral fusion by interacting with the viral gp41 NHR to form a heterotrimer, thus disrupting the formation of homotrimers. Therefore, N36^Mut(e,g)^ is about 50-fold more potent than its parent peptide N36 in inhibiting HIV-1 Env-mediated cell-cell fusion [38,41]. 

Although the above-mentioned peptides play a vital role in treating HIV/AIDS patients who fail to respond to the current anti-HIV drugs, further applications of these peptide-based fusion/entry inhibitors are significantly limited by their disadvantages, including the high cost of producing peptide and lack of oral bioavailability. Therefore, small-molecule HIV-1 fusion/entry inhibitors targeting the HIV-1 gp41 pocket with oral availability should be developed. 

## 3. Computer Modeling-Based Virtual Screening of HIV-1 Fusion Inhibitors Targeting gp41 Pocket

### 3.1. Introduction of Computer-Aided Molecular Docking Techniques

Computer-aided drug design (CADD) has become an important method in new drug research and development. It has a wide range of applications, including the drug discovery pipeline, from target identification to lead discovery and from lead optimization to preclinical study and even clinical trials. The molecular docking technique, or virtual screening, has become a promising technique in drug discovery. One such technique is ligand-based drug design, which depends on the knowledge of other molecules binding to the biological target of interest. Another technique is structure-based drug design, which relies on the knowledge of the three-dimensional structure of the biological target, which is obtained through methods such as X-ray crystallography or NMR spectroscopy. 

The combination of biophysical and biochemical techniques has been extensively applied in drug development using virtual screening. Several docking programs, such as FlexX [42], GLIDE [43], GOLD [44], and DOCK [45], have been widely used for almost two decades. These programs can automatically generate many possible orientations and conformations of a putative ligand within a receptor pocket. For each ligand candidate, tens of thousands of orientations can be generated and scored [46,47]. Scoring functions have been used to estimate the potential binding affinity of a molecular structure in a certain conformation and pose. Scoring can be done by force field-based methods, techniques based on the Poission-Boltzmann equation, potentials of mean forces, free energy perturbation and simple linear approximations [47]. 

### 3.2. Identification of ADS-J1 and Derivatives from ComGenex Database Using the Virtual Screening Program DOCK3.5

As mentioned above, the deep hydrophobic pocket, which, is located in the grooves of the gp41 NHR-trimer, accommodates three conserved hydrophobic residues (W628, W631, and I635) in the gp41 CHR region [48,49,50]. As such, it is also an important target for small molecule HIV-1 fusion inhibitors [8,9]. Therefore, we utilized the gp41 pocket as a target and a docking program, termed DOCK3.5, as a virtual screening tool to examine the ComGenex database of a chemical library consisting of 20,000 compounds. Briefly, we first created the molecular surface of the target site (gp41 pocket) and identified the important residues for possible interaction with the ligand molecule. Then, to fill the active site, we generated spheres that serve as a guide to locate ligands whose interatomic distance matches the intersphere-center distance and a grid box encompassing the spheres to save the steric and electrostatic information at each grid point so that the ligand orientation could be scored during docking. Subsequently, we searched thousands of orientations of ligands to match the center of the spheres and evaluated the ligand orientation by shape or force field scoring function. Finally, we localized local minima by simplex minimization [3,51,52]. 

**Figure 3:**
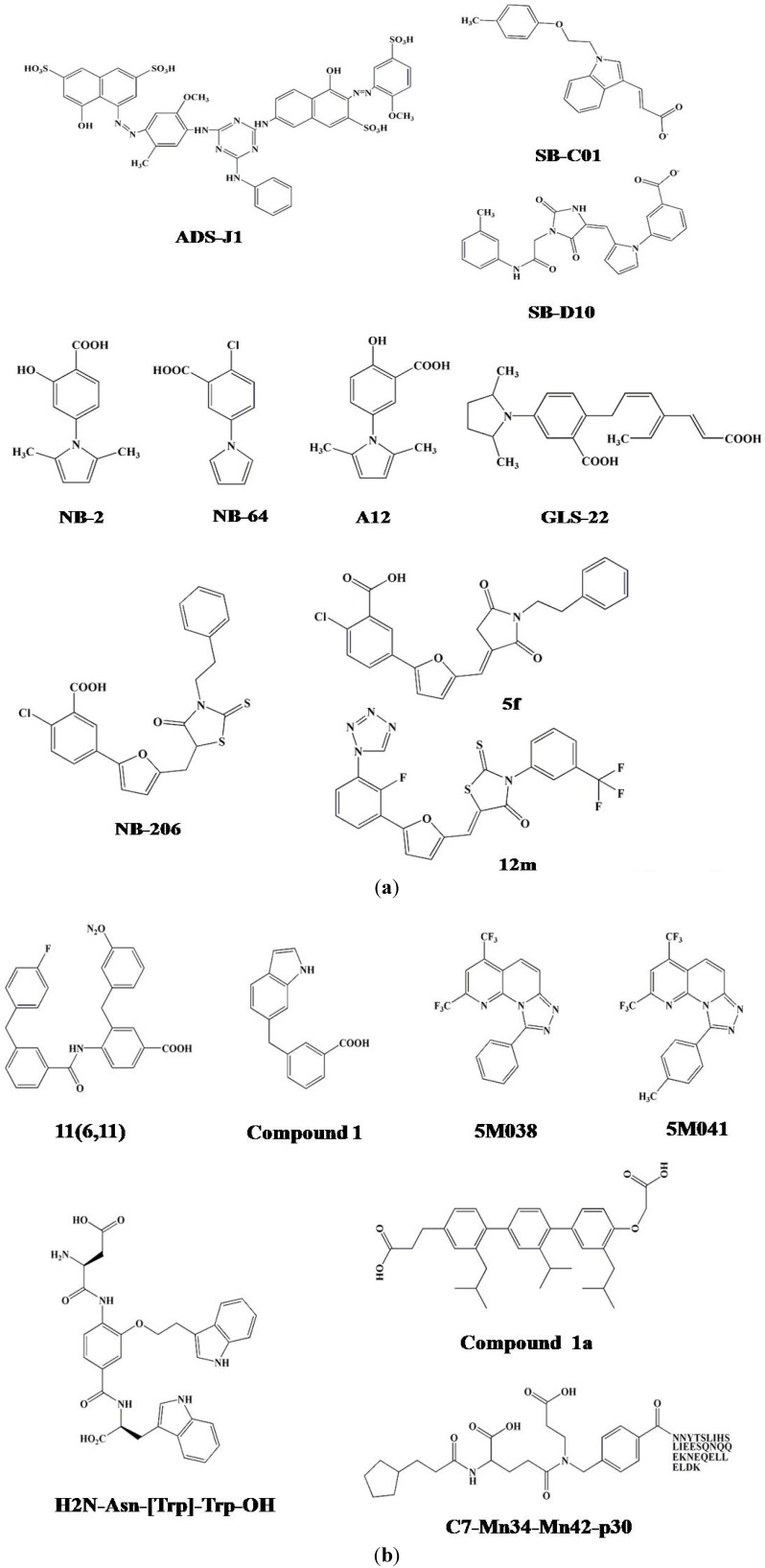
The chemical structures of the small molecule HIV-1 entry inhibitors targeting the gp41 pocket.

After screening, we selected 16 commercially available compounds with the highest docking score to test their inhibitory activity on the gp41 6-HB formation and their anti-HIV-1 activity, using a sandwich enzyme-linked immunosorbent assay (ELISA) with a 6-HB-specific monoclonal antibody (MAb), NC-1 [51,53]. We found that two of these compounds, 7-[6-phenylamino-4-[4-[(3,5–disulfo–8-hydroxynaphthyl)azo]–2–methoxy–5-methylphenylamino]-1,3,5–triazine–2-yl]–4–hydroxyl–3-[(2–methoxy–5-sulfophenyl)azo]–2-naphthalene sulfonic acid (ADS-J1) and 5-[(4–chloro–6–phenylamin-1, 3, 5–triazine–2-yl)-amino]–4–hydroxyl–3-[(4–methyl–6-sulfophenyl)azo]-2,7-naphthalene disulfonic acid (ADS-J2), could effectively block 6-HB formation and inhibit HIV-1-mediated cell fusion and cytopathic effect (Figure 3a, Table 1) [51]. 

Stereographic analysis indicates that the hydrophobic groups (phenyl and naphthalene) of ADS-J1 could interact with the hydrophobic residues (L568, V570 and W571) in the pocket **(Figure 1**C). One of the sulfonic acid groups of ADS-J1 is in close proximity to K574, a basic residue in the NHR region that locates around the pocket. Thus, the binding of ADS-J1 and K574 may block the interaction between K574 and D632, an amino acid in the C-Helix of gp41, to form a salt bridge. The above data suggest that ADS-J1 is a potent HIV-1 fusion/entry inhibitor. It is likely that ADS-J2 has lower activity than ADS-J1 by its inability to interact with the hydrophobic pocket residues, even though it binds to K574 [51]. The high molecular weight of ADS-J1 may also contribute to the prevention of 6-HB complex formation. It should be noted that ADS-J13 ranks higher than ADS-J1 in force field scoring, but it has no anti-HIV-1 activity [3,51]. Close visual inspection showed that ADS-J13 has no acidic group and cannot interact with K574 or any other charged residues, suggesting that acidic groups and charged groups, in addition to hydrophobic residues, play important roles in interacting with anti-HIV-1 drugs targeting the HIV-1 gp41 pocket.

Detailed mechanistic study reveals that ADS-J1 blocks the formation of the NC-1 detectable complex between N- and C-peptides, confirming that ADS-J1 inhibits HIV-1 infection by binding to the HIV-1 gp41 pocket [52]. Time-of-addition and time-of-removal studies (addition of an inhibitor to or removal of an inhibitor from the culture system at indicated time-points before or after viral infection) suggest that ADS-J1 inhibits HIV-1 replication in the early stage of entry and interacts with the HIV-1-infected cells rather than the targeted cells in the inhibition of cell-cell fusion [15]. A sCD4-based ELISA indicates that ADS-J1 cannot block gp120-CD4 binding, while another cell-based ELISA shows that it has weak interaction with CXCR4, an HIV-1 co-receptor. However, native polyacrylamide gel electrophoresis (N-PAGE) and circular dichroism (CD) analyses demonstrate that ADS-J1 can inhibit fusion-active gp41 core formation. IQN17 is a trimeric peptide that conjugates the GCN4 sequence (IQ) with a short NHR peptide (N17) involved in the formation of the gp41 hydrophobic pocket. By surface plasmon resonance assay, it was found that ADS-J1 could also bind directly to IQN17, resulting in the conformational change of IQN17 and the blockage of its interaction with a short D peptide, PIE7 [15,54]. 

These mechanistic studies suggest that ADS-J1 is an HIV-1 fusion inhibitor targeting gp41 with a binding site located in the NHR pocket region. In addition, other reports showed that ADS-J1, when used at a high concentration, could bind to the V3 loop in gp120 through its sulfonate groups [55,56]. 

### 3.3. Identification of SB-D10, SB-C01 and their Derivatives from the ZINC Database Using the Virtual Screening Program DOCK6.5

Similar to the screening of ADS-J1, a virtual screening against the gp41 deep pocket was recently performed [57]. Using the DOCK6.5 program, *ca.* 500,000 compounds were initially screened from the ZINC database [58,59,60]. Four key side chains of native gp41 C-helix, Trp117, Trp120, Asp121 and Ile124, were considered as the gp41 reference interacting with the conserved hydrophobic pocket. The van der Waals (VDW) footprint derived from the X-ray structure of 1AIK was used to identify compounds from the database that have similar footprint overlap [48,57]. 

All compounds were first flexibly docked to the gp41 receptor grid following the DOCK FLX protocol. After calculating the footprint similarity (FPS) score, 100,000 molecules containing the lowest-energy pose were retained. These molecules were then clustered by combining MACCS fingerprints with the Tanimoto coefficient of 0.75 in the MOE program [57]. The best scoring member of each cluster was retained using standard DOCK score (DCEVDW+ES), van der Waals footprint similarity score (FPSVDW), electrostatic footprint similarity score (FPSES), and the combined footprint sum (FPSVDW+ES) scoring methods. The resultant cluster heads were ranked accordingly, and among them, 500 top-ranked molecules were finally obtained [57]. A total of 115 compounds were purchased for further testing based on a selection protocol, of which 18 showed binding *K_i_* value <15 μM in a fluorescence-based binding assay. The inhibitory ability of these selected compounds was detected by cell-cell fusion and cytotoxicity assays, and seven of these had favorable properties, such as, low *K_i_*, low IC_50_, and high CC_50_ values. One of the compounds, termed SB-D10 (Figure 3a), contained an N-substituted pyrrole group similar to the structure of NB-2 and NB-64 inhibitors [14], but its size and layout were similar to those of NB-206 and its derivatives [61]. Another compound, SB-C01 (Figure 3a), which contained an indole group similar to some reported gp41 inhibitors [57,62], had striking overlap between its carboxylic acid and that of Asp 121. 

## 4. ELISA- and FLISA-Based High-Throughput Screening (HTS) Assays for Identification of HIV-1 Fusion Inhibitors Targeting gp41 Pocket

The *in vitro* model systems were initially established to imitate gp41 6-HB formation for the screening of fusion inhibitors targeting gp41. These model systems essentially detect whether or not the selected compound can inhibit the gp41 6-HB formation between an N-peptide (*e.g.*, N36) and a C-peptide (*e.g.*, C34). 

### 4.1. Sandwich ELISA, Direct ELISA, and Fluorescence-Linked Immunosorbent Assay (FLISA)

We first developed a sandwich ELISA to screen inhibitors that block gp41 6-HB formation [63]. The plate is coated with a rabbit polyclonal antibody against the HIV-1 gp41 N- and C-peptides, followed by addition of the mixture of an N-peptide, N36, the inhibitor (or PBS), and a C-peptide, C34. The bound 6-HB is then detected by addition of the 6-HB-specific mAb NC-1 [64], and the inhibitory activity of the inhibitor on the 6-HB formation is measured using an ELISA reader. 

Next, we developed a direct ELISA on the basis of the sandwich ELISA [47,65]. In this assay, the plate is coated with the 6-HB-specific mAb NC-1 before addition of the mixture of N36 peptide and biotin-labeled C34. After removal of unbound peptides and addition of streptavidin-conjugated HRP and the substrate sequentially, inhibitory activity on 6-HB formation is measured using an ELISA reader. Compared with the sandwich ELISA, the direct ELISA is more suitable for HTS because all incubation steps are conducted at room temperature, while for the sandwich ELISA, all incubation steps must be performed at 37 ºC. Moreover, sandwich ELISA requires frequent transportation of the plates in and out of the 37 ºC incubators, which may result in higher experimental variation. In addition, direct ELISA typically requires only half the processing time (about 3 hours) compared to sandwich ELISA, thus saving time in a cost effective manner. The above findings suggest that direct ELISA is more rapid, convenient and economical, thus more suitable for HTS.

Subsequently, we further developed an FLISA by using C34-FITC to replace C34-biotin in the direct ELISA [47,65]. Without the need to add streptavidin-conjugated HRP and the substrate, inhibitory activity on 6-HB formation is detected by measuring the fluorescence intensity using a multifunctional reader. While FLISA shares specificity and sensitivity similar to the sandwich and direct ELISAs, it is more convenient, rapid, and economical than the ELISA assays because fewer experimental steps and reagents are required for FLISA. 

We then used the HTS, as described above, to screen a chemical library consisting of 30,040 “drug-like” compounds. We found that two of these compounds, NB-2 and NB-64, both of which belong to N-substituted pyrrole derivatives (Figure 3a), could significantly inhibit 6-HB formation and HIV-1-mediated cell-cell fusion [14]. They inhibited infection by a series of primary HIV-1 isolates, including clades A to G and group O, and laboratory-adapted HIV-1 strains, such as IIIB, RF, SF2, and AZT-R, at low micromolar levels (Table 1) [14]. However, these two compounds could also lose anti-HIV-1 activity upon loss of their COOH group, suggesting that the COOH group plays an important role in mediating antiviral activity. Using a computer-aided molecular docking technique, we further analyzed the interaction of these two compounds with the amino acid residues in the gp41 pocket and surrounding area, and we found that either NB-2 or NB-64 could form a salt bridge between their COOH group, respectively, and a positively charged residue K574, thus blocking 6-HB formation and subsequently inhibiting HIV-1-mediated membrane fusion. 

The identification of NB-2 and NB-64 prompted us to modify their structures to develop new lead compounds with improved anti-HIV-1 activity. We designed and synthesized 42 *N*-carboxyphenylpyrrole derivatives in two categories (A and B series), including *N*-phenylpyrrole and *N*-phenyl-2,5-dimethylpyrrole derivatives (A1-A10 and A11-A20, respectively), and carboxyphenyl compounds with a ring consisting of 1,2,4-oxadiazole (B1-B11), thiadiazole (B12), maleimide (B13-B15), or rhodamine (B16-B22) [66]. We found that half of the A series compounds exhibited anti-HIV-1 activity at low micromolar level, but only two of the B series displayed moderate inhibitory activity. Among the A series compounds, A12 is the most effective in inhibiting p24 production and gp41 6-HB formation (Table 1). 

A novel drug design methodology, GeometryFit, was then used to modify the structure of A12 (Figure 3a), and based on this restructuring, five new compounds were designed and synthesized. Two of these newly designed compounds, GLS-22 and GLS-23, showed better inhibitory activity on 6-HB formation, HIV-1-mediated cell-cell fusion and HIV-1 replication than A12 because they target more contact motifs within the gp41 pocket (Table 1) [67]. 

Next, we designed and synthesized a series of 2-aryl 5-(4-oxo-3-phenethyl-2- thioxothiazolidiny- lidenemethyl) furans with higher molecular size (437-515 Da), hoping to occupy more space in the deep hydrophobic pocket on the gp41 NHR trimer. Among the 15 compounds tested, we found that three of them, 11a (NB-293), 11b, and 11d (NB-206), exhibited antiviral activity at the nanomolar level against both primary and laboratory-adapted HIV-1 strains (Figure 3a, Table 1) [68]. 

By modifying the chemical structure of NB-206, we designed and synthesized a series of 5-((arylfuran/1H-pyrrol-2-yl)methylene)-2-thioxo-3-(3-(trifluoromethyl)phenyl) thiazolidin-4-ones (12a-o) and 2,5-disubstituted furans/pyrroles (5a–h) [69,70]. We found that most of the 12a-o compounds showed improved inhibitory activity on 6-HB formation and HIV-1 replication. Two of them, 12l and 12m, exhibited potent anti-HIV-1 activity at low nanomolar level and high selectivity indexes (SI: >2000) (Figure 3a, Table 1). However, the 2,5-disubstituted furans/pyrroles (5a–h) showed much lower anti-HIV-1 activity than NB-206 (Figure 3a, Table 1) [69,70].

## 5. Metal-Ion-Based NHR-Trimer as the Target for Screening of HIV-1 Fusion Inhibitors Targeting gp41 Pocket

As previously described [71], the transiently exposed NHR-trimeric conformation at the fusion-intermediate state is very unstable, and the N-peptides have a tendency to aggregate under physiological condition [10]. Therefore, it is very challenging to establish an HTS assay using the transiently exposed NHR coiled-coil in solution [72,73]. To resolve this problem, several strategies were employed, including the design of 5-Helix protein [74], the addition of a soluble trimeric motif, GCN4 or foldon, to stabilize the coiled-coil [75,76], the covalent linking of the peptides of the coiled coil [77], or the usage of N-terminal ferrous ion ligation to design a stable NHR-trimer [72,73,78,79]. 

Gochin and colleagues used the fluorescence resonance energy transfer (FRET) technique and a metal-ion-based NHR-trimer as a target to screen for HIV-1 fusion inhibitors targeting gp41 core [72,73,78,79]. This HTS assay is designed on the basis of competitive inhibition between the NHR complex ([Fe(II)(env2.0)_3_]^2+^ as the FRET acceptor and a modified C-peptide (C18-Aib-LY) as the FRET donor. The ([Fe(II)(env2.0)_3_]^2+^ complex was designed by adding Fe(II) to a Tris–2,2’-bipyridine-5-carboxypeptide complex to form a tris-bipyridyl metal complex, which was linked to the N-terminus of a 31-mer N-peptide containing the pocket-forming sequence. C18-Aib-LY was designed by labeling an 18-mer C-peptide, C18, which contains a PBD with a fluorophore, lucifer yellow iodoacetamide (LY). This HTS assay, which is performed by mixing acceptor and donor peptides with the compounds to be tested and measuring fluorescence intensity, exhibits high integrity, specificity and sensitivity. By screening several series of small molecule compounds with this HTS assay, they have identified a number of HIV-1 fusion inhibitors, such as 11(6,11) and compound 1, having inhibitory activity with IC50 at low µM level in HIV-1 Env-mediated cell-cell fusion assays (Figure 3b and Table 1) [73,80]. 

## 6. Five-Helix-Based HTS Assay to Screen HIV-1 Fusion Inhibitors Targeting gp41 Pocket

As mentioned above, the polypeptide 5-Helix is a potent HIV-1 fusion inhibitor targeting the gp41 CHR region [40]. Because of its exposed groove and the hydrophobic pocket, 5-Helix can also be used as a target for screening HIV-1 fusion/entry inhibitors [81]. Using gp41-5, a 5-Helix-like soluble and single-chain protein, as a target, Frey *et al.* developed a fluorescence polarization (FP)-based HTS assay to screen small molecule HIV-1 fusion inhibitors [82]. They identified two small molecule HIV-1 fusion inhibitors, designated 5M038 and 5M041, from several chemical libraries consisting of 38,400 compounds (Figure 3b). Experimental data indicate that these two compounds inhibited HIV-1 Env-mediated cell-cell fusion and HIV-1 infection at the micromolar level (Table 1). Because of the extremely high binding affinity between 5-Helix and a C-peptide (*K_d_* =~0.5 pM) [81], the 5-Helix-based HTS assay may not be suitable for the primary screening of lead compounds with low gp41 pocket-binding affinity.

## 7. Alpha-Helix Mimicry-Based Design or HTS Assay for Identification of HIV-1 Fusion Inhibitors Targeting gp41 Pocket

It has been shown that both the NHR and CHR in the 6-HB maintain α-helical conformation. The interaction of the CHR with the NHR hydrophobic pocket is mediated by four critical amino acid residues, including Trp^628^, Trp^631^, Asp^632^ and Ile^635^, on the CHR-helix. Residue Asp^632^ electrostatically interacts with Lys^574^ at the pocket periphery [83]. The 3,2',2''-terphenyl derivative has been reported as an effective mimic of the surface functionality of an α-helix [84]. Based on these findings, a molecular scaffold was thus designed to mimic the surface of an α-helix to screen HIV-1 fusion inhibitors targeting the gp41 pocket. Using the 3,2',2''-terphenyl derivative as a parent structure, one terphenyl derivative compound, **1a,** was designed to mimic the side chains of an *i*, *I* + *4*, *I* + *7 dad* hydrophobic surface. We found that compound **1a** could inhibit HIV-1-mediated cell-cell fusion and 6-HB formation at low μM concentration [83].

Most recently, Whitby *et al.* have established a comprehensive α-helix mimetic library for binding the gp41 NHR hydrophobic pocket [85]. In this template, 20 natural amino acids were substituted on the three positions of the side chains of the α-helix. Together with all combinations, an 8000-member library (20 × 20 × 20) was created in order to identify the combinations that could bind with the gp41 hydrophobic pocket and exhibit effective inhibitory activity against HIV-1. Using a step-by-step approach, they found that three of the α-helix mimetics, H2N-Trp-[Trp]-Leu-OH, H2N-Asn-[Trp]-Trp-OH, and H2N-TyrMe-[Trp]-Trp-OH, exhibited effective activity against HIV-1 Env-mediated cell–cell fusion with IC50 values ranging from 5 to 8 μM (Figure 3b, Table 1) [85].

Ferrer *et al.* used another approach to identify gp41 pocket-binding α-helix mimetics [86,87]. They first created a combinatorial library of 61,275 compounds using split-pool synthesis compatible with recursive deconvolution. Here, each compound consists of three building blocks, cap (C), monomer 1 (M1) and monomer 2 (M2), which are then linked to the N-terminus of C-peptide C30 (residues 636-665, corresponding to the gp41 CHR without the PBD) in anticipation of allowing the C30 peptide to bring the small molecule compound to the gp41 pocket. Using a colorimetric, affinity-based selection assay and cell-cell fusion assay, they found that one of the hybrid molecules, C7-Mn34-Mn42-P30 (445 Dalton), exhibited inhibitory activity against HIV-1 Env-mediated cell-cell fusion with an EC_50_ of 300 nM (Figure 3b). Although it is unknown whether the small organic compound C7-Mn34-Mn42 itself has any anti-HIV-1 activity [86], X-ray crystallographic analyses showed that it could bind to the gp41 pocket in the gp41 core [87]. But unexpectedly, this moiety binds in two models, each with about 50% occupancy, possibly resulting in its low binding affinity to the gp41 pocket.

**Table 1:** The properties of the small molecule HIV-1 fusion inhibitors targeting gp41 pocket.

Inhibitors	IC_50 _(μM)			CC_50_	SI	Ref.
	Inhibiting 6-HB formation	Inhibiting p24 production	Inhibiting Cell-cell fusion			
Computer modeling-based virtual screening						
ADS-J1	0.73	8.29	4.95	292.16	35.24	[51]
ADS-J2	3.18	30.76	21.85	289.94	9.43	[51]
SB-D10		160		>100		[57]
SC-01		43		>50		[57]
ELISA- or FLISA-based high-throughput screening (HTS) assays						
NB-2		1.04	6.74	2755		[14]
NB-64	58.74	2.21	29.92	>4000		[14]
A12	29.39-37.36	0.69-28.19	43.24	133.5-333.8		[66]
GLS-22	20.73	4.91	3.60	255.28		[67]
GLS-23	21.75	8.30	8.30	227.27		[67]
NB-293(11a)		0.098		19.35	440	[68]
**11b**		0.031		42.14	426	[68]
NB-206 (**11d**)		0.017		16.82	330	[68]
**12l**		0.018-0.32		66.34	3686	[70]
**12m**		0.014-0.99		27.85	1989	[70]
**5f**	15.55	6.90	43.55	118.45		[69]
**5h**	12.89	16.79	45.92	>320		[69]
Fluorescence resonance energy transfer (FRET) using metal ion						
11(6,11)		8				[73]
Compound **1**	3.2			>500		[80]
Fluorescence polarization assay						
5M038		19	38			[82]
5M041		18	18			[82]
alpha-helical mimicry						
Compound 1a	13.18		15.70			[83]
H2N-Asn-[Trp]-Trp-OH			6			[85]
H2N-Trp-[Trp]-Leu-OH			5			[85]
H2N-TyrMe-[Trp]-Trp-OH			8			[85]
Structure-based combinatorial approach						
C7-Mn34-Mn42-p30			0.3			[86]

## 8. Synergistic Combinations of Multiple HIV Entry Inhibitors

It has long been proven that combining antiretroviral agents with different mechanisms, such as reverse transcriptase inhibitors and protease inhibitors, which are the major components of HAART, is the most effective therapeutic option to control of HIV-1 replication, especially against HIV-1 variants with multi-drug resistance [88]. The entry of HIV-1 into the target cell is a multi-step process, thereby providing a number of targets for HIV-1 entry inhibitors. Therefore, combining HIV entry inhibitors that target at the different proteins involving HIV entry, such as viral gp120 and gp41, as well as the cellular receptor CD4 or coreceptor CCR5 and CXCR4, is expected to have synergistic effect. 

PRO 542 is a gp120-targeted HIV-1 entry inhibitor by blocking viral attachment to CD4 cells. Combination of PRO 542 with T20 resulted in synergistic inhibition of virus-cell and cell-cell fusion, with more than 10-fold dose reductions [89]. Combining T20 with AMD3100 (a CXCR4 antagonist) or SCH-C (a CCR5 antagonist) exhibited strong synergistic anti-HIV-1 activity [90,91]. These findings indicate that combinational use of the HIV entry inhibitors with different mechanisms may further improve the antiretroviral therapy because of the increased efficacy and decreased toxicity, as well as overcoming drug resistance. 

Interestingly, we found that combinations of the gp41 CHR peptide-based HIV entry inhibitors targeting different regions in the gp41 NHR also exhibited potent synergistic anti-HIV-1 activity. For example, combination of enfuvirtide (T20), which binds to the N-terminal region of NHR (containing the GIV motif) but lacks the pocket-binding domain, with sifuvirtide or T1144 (both contain the pocket-binding domain) resulted in strong synergistic antiviral activity against a broad spectrum of HIV-1 strains, especially those resistant to T20 [92,93]. Based on this result, we designed a chimera peptide consisting of T20, a 35-mer linker and T1144, designed TLT35, which showed high potency against HIV-1 gp41-mediated cell-cell fusion and HIV-1 infection [94]. However, the anti-HIV-1 activity of TLT35 was lower than that of the T20/T1144 combination, suggesting that some of the synergistic anti-HIV-1 effect otherwise observed from the combinational use of the isolated peptides T20 and T1144 may be abolished by the covalent linkage between the two peptide sequences in TLT35, which enhances the interaction between T20 and T1144, resulting in the shielding of their NHR-binding sites [95]. 

## 9. Conclusions

The HIV-1 Env gp41 mediates viral fusion and entry into the target cell. The hydrophobic pocket in the exposed grooves on the gp41 NHR-trimer plays an important role in stabilizing the gp41 6-HB and serves as an attractive target for identification of peptide- and small molecule compound-based HIV-1 fusion/entry inhibitors. A series of HTS assays based on the gp41 pocket as the target have been developed to screen for HIV fusion/entry inhibitors. T-20 peptide was the first HIV entry inhibitor approved by the U.S. FDA to treat HIV/AIDS patients who fail to respond to current antiretroviral drugs. Several C-peptides containing the PBD with improved anti-HIV-1 potency, half-life, and drug-resistant profiles have been developed in preclinical and clinical studies. A number of small molecule compounds with HIV-1 fusion inhibitory activity at nM or low µM, such as NB-2, NB-206, 11(6,11), 5M041, and H2N-Trp-[Trp]-Leu-OH, have been identified as leads. 

It is expected that one of these leads will be further developed as a novel anti-HIV-1 drug for use to treat patients who fail to respond to the current antiretrovirals, such as the RTIs and PIs. Therefore, the small molecule HIV-1 entry inhibitors targeting gp120 and gp41 will be a critical addition to the as an important component in highly active antiretroviral therapy (HAART). To avoid the emergence of drug resistance, HIV entry inhibitors, like the anti-HIV drugs from other classes, should not be used as monotherapy. Because of the promising synergistic antiviral effect of combining HIV entry inhibitors targeting different proteins involving in HIV fusion/entry, these inhibitors may be used in combination in clinics for treating infection by HIV-1 strains that are highly resistant to the current antiretrovirals. The challenges for further development of the HIV entry inhibitors include improving their pharmacokinetic (PK) and druggable properties, such as metabolic stability and non-specific toxicity, and reducing the production cost and making them easy to use. Hopefully, a small molecule HIV entry inhibitor will become an essential component of the once daily single tablet regimen (STR), like the one, emtricitabine (FTC)/rilpivirine (RPV)/tenofovir (TDF), as the patients’ preferable treatment option. 

Furthermore, the approaches for identifying HIV fusion/entry inhibitors can also be applied for discovery of viral entry inhibitors against other enveloped viruses with type I membrane fusion proteins.
